# Early Detection of Lymph Node Metastasis Using Primary Head and Neck Cancer Computed Tomography and Fluorescence Lifetime Imaging

**DOI:** 10.3390/diagnostics14182097

**Published:** 2024-09-23

**Authors:** Nimu Yuan, Mohamed A. Hassan, Katjana Ehrlich, Brent W. Weyers, Garrick Biddle, Vladimir Ivanovic, Osama A. A. Raslan, Dorina Gui, Marianne Abouyared, Arnaud F. Bewley, Andrew C. Birkeland, D. Gregory Farwell, Laura Marcu, Jinyi Qi

**Affiliations:** 1 Department of Biomedical Engineering, University of California, Davis, CA 95616, USA; nmyuan@ucdavis.edu (N.Y.); mahmohamed@ucdavis.edu (M.A.H.); kehr@ucdavis.edu (K.E.); brentwweyers@gmail.com (B.W.W.); 2 Department of Radiology—Neuroradiology, University of California, Davis, CA 95817, USA; gbiddle@ucdavis.edu (G.B.); oraslan@ucdavis.edu (O.A.A.R.); 3 Department of Neurology, University of California, Davis, CA 95817, USA; vivanovic@ucdavis.edu; 4 Department of Pathology and Laboratory Medicine, University of California, Davis, CA 95817, USA; dgui@ucdavis.edu; 5 Department of Otolaryngology—Head & Neck Surgery, University of California, Davis, CA 95817, USA; mabouyared@ucdavis.edu (M.A.); abewley@ucdavis.edu (A.F.B.); acbirkeland@ucdavis.edu (A.C.B.); 6 Department of Otorhinolaryngology–Head and Neck Surgery, University of Pennsylvania, Philadelphia, PA 19104, USA; greg.farwell@pennmedicine.upenn.edu; 7 Department of Neurological Surgery, University of California, Davis, CA 95817, USA

**Keywords:** radiomics, machine learning, head and neck cancer, lymph node metastasis, CT, fluorescence lifetime imaging

## Abstract

**Objectives**: Early detection and accurate diagnosis of lymph node metastasis (LNM) in head and neck cancer (HNC) are crucial for enhancing patient prognosis and survival rates. Current imaging methods have limitations, necessitating new evaluation of new diagnostic techniques. This study investigates the potential of combining pre-operative CT and intra-operative fluorescence lifetime imaging (FLIm) to enhance LNM prediction in HNC using primary tumor signatures. **Methods**: CT and FLIm data were collected from 46 HNC patients. A total of 42 FLIm features and 924 CT radiomic features were extracted from the primary tumor site and fused. A support vector machine (SVM) model with a radial basis function kernel was trained to predict LNM. Hyperparameter tuning was conducted using 10-fold nested cross-validation. Prediction performance was evaluated using balanced accuracy (bACC) and the area under the ROC curve (AUC). **Results**: The model, leveraging combined CT and FLIm features, demonstrated improved testing accuracy (bACC: 0.71, AUC: 0.79) over the CT-only (bACC: 0.58, AUC: 0.67) and FLIm-only (bACC: 0.61, AUC: 0.72) models. Feature selection identified that a subset of 10 FLIm and 10 CT features provided optimal predictive capability. Feature contribution analysis identified high-pass and low-pass wavelet-filtered CT images as well as Laguerre coefficients from FLIm as key predictors. **Conclusions**: Combining CT and FLIm of the primary tumor improves the prediction of HNC LNM compared to either modality alone. Significance: This study underscores the potential of combining pre-operative radiomics with intra-operative FLIm for more accurate LNM prediction in HNC, offering promise to enhance patient outcomes.

## 1. Introduction

Head and neck cancer (HNC), which arises from the mucosal surfaces of the oral cavity, pharynx, larynx, and related structures, accounts for a significant portion of global cancer diagnoses [1,2]. According to the latest official statistic from 2020 [3], mucosal HNC is the 8th most common cancer worldwide. A particularly alarming aspect of these malignancies is their inherent ability to spread or metastasize to regional lymph nodes, adversely affecting prognosis and oncologic outcomes.

Precise diagnosis and therapeutic management of cervical nodal metastasis is crucial, as lymph node metastasis (LNM) serve as the most significant prognostic indicator of overall survival in patients with HNC [4,5]. Early assessment and detection of LNM allows for prompt and effective treatment interventions, which can significantly increase survival rates [6]. In comparison, late detection of LNM significantly decreases survival rates; the presence of a single positive lymph node reduces survival by up to 50% [6,7].

Detecting LNM in HNC is essential for effective management and treatment planning, yet it is riddled with complexities due to several factors. Patients with early lymph node involvement may remain asymptomatic and may not have clinically or radiographically detectable disease, which delays diagnosis and intervention [1,8]. Pre-operative determination of the status of the lymph nodes may require biopsy, most commonly with a fine needle. This minimally invasive technique can be problematic due to anatomic constraints, sample error, and a low but possible risk of complications [1,9]. Meanwhile, conventional imaging modalities, such as CT and magnetic resonance imaging (MRI), while invaluable, sometimes struggle to differentiate between inflammatory lymph nodes and those harboring metastatic cells, especially when the nodes are not significantly enlarged and, more specifically, when the short-axis diameter of the lymph node is less than 1 cm [10,11,12,13]. Therefore, the accuracy of lymph node identification is highly dependent on the experience of the radiologist and the unique characteristics of the lymph node. In support of this point, D’Cruz et al. have indicated that the rate of positive lymph nodes in clinically and radiologically normal necks may reach up to 29%, underscoring the necessity for more advanced techniques to accurately evaluate the risk of LNM [6]. Lastly, the heterogeneity of HNC, where primary tumors of the same size and location can behave differently, poses another layer of challenge [1,9]. As a result of these challenges, current recommendations include sentinel lymph node biopsy (SLNB) or diagnostic lymph node dissection in patients with indeterminate pre-operative lymph node assessments and primary tumors at sufficient risk for cervical lymph node metastasis. However, SLNB is still controversial and being studied [14] and elective neck dissections can carry risk and morbidity, e.g., potential nerve injury, impairment in post-operative function, increased operative times, and the risk of bleeding [15]. These concerns suggest an opportunity for the continued evolution of diagnostic methodologies.

In recent years, radiomics of conventional imaging modalities has been increasingly utilized for the detection of LNM in HNC [16]. Machine learning algorithms can extract and analyze numerous radiomic features within medical imaging data, which can be useful for detecting LNM in HNC. Giannitto et al. explored the potential of radiomics-based machine learning to identify pathological lymph nodes in HNC, indicating a growing utilization of machine learning in detecting lymph node metastases [17]. Wang et al. focused on radiomics-based MRI for the pre-operative prediction of LNM, emphasizing the high predictive value of multiomic signatures derived from MRI radiomics, the apparent diffusion coefficient, and the lymph node size for determining the status of cervical lymph nodes [18]. Zhai et al. aimed to develop a pre-treatment radiomics-based prediction model to identify pathological lymph nodes at risk of failure post-definitive radiotherapy using radiomic features obtained from pathological lymph nodes that are crucial for tailoring treatment plans for patients with head and neck squamous cell carcinoma [19]. In another study, Chen et al. proposed a deep-learning-based network to extract features from CT and PET images and then to predict LNM [20]. Additionally, the HECKTOR challenge 2022 (Task 1) focused on the automatic segmentation of head and neck primary tumor and lymph nodes in FDG-PET/CT images, further revealing the potential relevance of HNC and LNM prediction [21,22]. These prior studies exemplify the feasibility of using radiomic features to predict LNM. However, these studies focus on lymph node features and cannot identify positive lymph nodes that appear normal radiographically. To address this limitation, we leverage radiomic features of the primary tumor in this study.

Label-free fluorescence lifetime imaging (FLIm) has evolved as an intra-operative tool for tumor margin detection in HNC surgery [23]. FLIm can identify tissue composition by measuring fluorescent signatures from endogenous fluorophores. Our previous studies show that FLIm achieves high accuracy (AUC of 0.94) in differentiating healthy and cancer tissues [24]. These findings suggest that fluorescence decay characteristics may correlate with biological behavior and have the potential for identifying lymph node metastases. We hypothesized that a combination of radiomics and FLIm features of primary tumors could improve the accuracy of LNM prediction. This study aims to investigate the potential of integrating fluorescence signatures from intra-operative FLIm together with pre-operative CT images to improve LNM prediction. The results of this study could potentially be used for real-time guidance of HNC surgery based on LNM and tumor aggressiveness predictions. To our knowledge, this is the first study combining pre-operative CT imaging and intra-operative FLIm for surgical guidance. Ultimately, this approach has the potential to significantly impact the management and overall prognosis of HNC patients.

## 2. Materials and Methods

### 2.1. Study Population

This retrospective study was approved by the institutional review board. Patients aged 18 years or older with a pre-cancerous condition (e.g., high-grade dysplasia or leukoplakia) or carcinoma of the oropharynx or oral cavity who were diagnosed at the University of California, Davis Otolaryngology—Head and Neck Surgery department between June 2017 and December 2020 were included. Exclusion criteria were: (1) an inability to consent, (2) pediatric patients, (3) pregnant women, (4) prisoners, (5) stage M1 (distant metastasis) cancer, (6) HIV+ patients (for research personnel safety), (7) prior major head and neck surgery with significant scarring (which may confound FLIm measurements), (8) use of orally administered crack-cocaine or methamphetamine (MA) (which may confound FLIm measurements), (9) absence of a tumor on FLIm scans, and (10) absence of pre-operative CT scans. The final cohort consisted of 46 head and neck cancer (HNC) patients.

As shown in Table 1, among the 46 patients, the most common cancer locations were the oropharynx (56.5%), particularly the palatine tonsil (30.4%), the base of the tongue (15.2%), and the oral cavity (43.5%), with the superior tongue being the most frequent site (30.4%). Less common tumor sites included the gingiva (6.5%), floor of the mouth (4.3%), and palate (2.2%) in the oral cavity, as well as the pharynx (4.3%), retrimolar trigone (4.3%), and vallecula (2.2%) in the oropharynx. The gender split comprised 37 males (80.4%) and 9 females (19.6%). Cumulatively, 58.7% had positive LN and 41.3% had negative LN. The mean age was 63.3 ± 11.9 years.

### 2.2. Workflow

**Figure 1:**
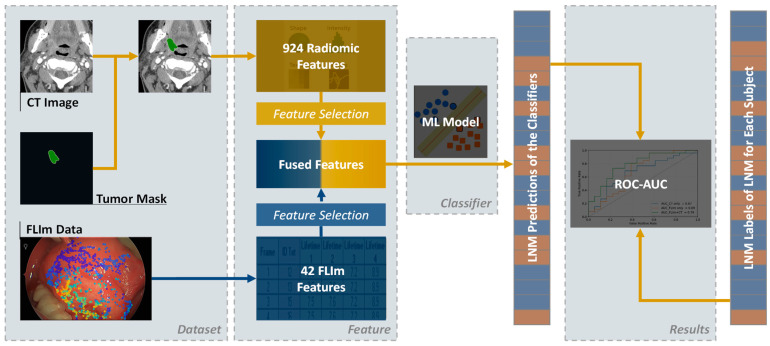
The overall workflow of predicting LNM using CT and FLIm. The original data used for predicting LNM are the CT and FLIm data. The primary tumor masks of HNC were manually delineated on CT images by an experienced radiologist. Primary tumors in CT images were used to extract radiomic features; FLIm features were extracted from three spectral channels for each point measurement. Two sets of features were generated separately and then fused together. The fused and selected features were further used for the ML model training, validation, and testing. Abbreviations: ML—machine learning, LNM—lymph node metastasis, ROC—receiver operating characteristic, AUC—area under curve.

Figure 1 shows the overall workflow for LNM prediction. The dataset comprised pre-operative CT scans for extracting radiomic features and intra-operative FLIm data acquired in vivo during tumor resection surgery. Primary HNCs were manually delineated on CT images by experienced radiologists, whereas the FLIm data were validated against primary tumor histopathology. Forty-two FLIm features were extracted from each FLIm measurement and then fused with the CT radiomic features. The fused features were fed into a machine learning (ML) model designed for the binary prediction of LNM, categorizing outcomes as positive or negative using surgical pathology from neck dissections as the ground truth.

### 2.3. Data Acquisition and Feature Extraction

#### 2.3.1. CT Data

Each patient underwent a pre-operative CT scan, with approximately 90% of the CT scans performed within three months preceding surgery. The CT data were sourced from over 15 institutions, using scanners from various manufacturers, and were subject to a wide range of scanning protocols and reconstruction parameters, including but not limited to dose level, spatial resolution, pixel size, and slice thickness. Sample CT images alongside FLIm data are illustrated in Supplementary Material Figure S1.

CT radiomic features were extracted following the image biomarker standardization initiative (ISBI) [25]. Specifically, CT images were re-sampled to 1.0 mm cubic voxels. All images were thresholded between −1000 and 1000 Hounsfield units (HU). Radiomic features were then computed from primary tumor regions delineated by Dr. V.I., who has 15 years of clinical experience in ENT (ear, nose, and throat) imaging. Automatic range re-segmentation [26] was used to remove boundary voxels outside the soft-tissue range (−200 to 200 HU), with final masks manually verified.

Radiomic features were extracted from both the original images and the derived images using different filters. Specifically, the filters included 3-D Laplacian of Gaussian (LoG) filters, with sigma values of 1.0, 2.0, 3.0, 4.0, and 5.0 mm and 2-D wavelet filters that included all possible combinations of applying either a high- or low-pass filter in each of the two dimensions, e.g., HL, HL, LH, and LL, where H indicates a high-pass and L indicates a low-pass. Seven major classes of the radiomic features were analyzed in this study: shape-based features (14 features and only based on tumor masks), first-order statistics (18 features), gray level co-occurrence matrix (GLCM; 22 features), gray level size zone matrix (GLSZM; 16 features), gray level run length matrix (GLRLM; 16 features), neighboring gray tone difference matrix (NGTDM; 5 features), and gray level dependence matrix (GLDM; 14 features). A total of 924 features were extracted. Detailed feature names can be found in Supplementary Material Figure S2.

#### 2.3.2. FLIm Data

A custom-made FLIm device was utilized for data collection in this study. The FLIm measurements were taken immediately before the tumor’s excision during surgery. Specific details regarding the hardware [27,28] and data processing [28,29] can be found in the corresponding references. Briefly, FLIm data were acquired by using a 355 nm pulsed excitation laser to induce tissue autofluorescence, averaging all measurements four times, resulting in 30 averaged readings every second. The mechanism operated on four spectral channels, each designed to match the autofluorescence emission maxima of various components, specifically targeting key markers for cancer such as collagen, nicotinamide adenine dinucleotides (NADHs), and flavin adenine dinucleotides (FADs). These components are crucial for the study’s focus of identifying cancerous tissues. The spectral channels were configured as follows: channel 1: 390 ± 20 nm, channel 2: 470 ± 14 nm, channel 3: 542 ± 25 nm, and channel 4: 629 ± 26.5 nm. The average FLIm scan lasted roughly 45 s, translating to approximately 1350 averaged point measurements per scanned surgical field. Scans were done before initiating surgical tissue removal. Generally, minimal to no blood was found in the surgical area. In instances of bleeding, the region was cleaned with isotonic saline and subsequently aspirated before optical scanning.

Data acquisition and visualization methods were customized as per the surgical context. A fiber probe was employed for transoral robotic surgeries (TORS), incorporating a 3D printed stainless steel grasper tab at the fiber probe’s end for better control via the da Vinci instruments; for the non-TORS surgeries, a Stryker endoscope and a handheld fiber probe with a stainless steel shaft and a ball lens at the distal end were used. The FLIm data were overlaid onto the endoscopic/robotic video of the surgical zone, assisting the surgeon during the data collection phase.

Spectral channels 1–3 were used for the analysis, while channel 4 was excluded due to its low signal-to-noise ratio. The raw FLIm waveforms underwent background subtraction. Laguerre-based deconvolution [29] was then applied to retrieve fluorescence decay characteristics for each channel, deriving the average lifetime from the Laguerre coefficients. This process generated 42 features: 3 average lifetimes, 3 intensities, and 36 Laguerre coefficients.

### 2.4. Model Training and Validation

#### 2.4.1. Conditional Random Grouping

In previous studies, all subjects were randomly divided into subsets. However, due to the limited number of subjects and the heterogeneity in tumor anatomy, random splitting may generate large variation between subsets because FLIm features of different anatomies may vary. In order to balance the distribution of anatomies across the subsets, the information on anatomical locations was taken into account during the grouping procedure. Conditional random grouping ensures that each subset contains four to five subjects, with one to two palatine tonsil subjects, one to two superior tongue subjects, one base of tongue subject, and one “other” type subject. In addition, care was taken to avoid subsets containing “all-positive” or “all-negative” LNM labels.

#### 2.4.2. Nested Cross-Validation

Figure S3 in the Supplementary Material illustrates the division of all 46 subjects into 10 subsets, D1–D10, as per the conditional random grouping criteria. Each subset was tested using the classifier trained on the other nine subsets. A nested 9-fold CV was employed on the training subsets to fine-tune the classifier’s hyperparameters.

#### 2.4.3. Weighted Samples

To address the issue of imbalance in the distribution of FLIm measurements across subjects, we employed a sample weighting strategy. Specifically, for both the FLIm-only and CT + FLIm classifiers, the weights were calculated as the reciprocal of the total number of FLIm measurements available for that patient. This approach ensures that all subjects, regardless of whether they have fewer or more FLIm measurements, can contribute equally to the model, thereby mitigating the effects of data imbalance.

#### 2.4.4. Feature Reduction and Selection

Features were normalized by removing the mean and dividing by the standard deviation before training; dimension reduction was performed by feature selection based on feature scores using the F-value of the analysis of variance (ANOVA) method [30] on training data only. The F-value is the ratio of the between-group variation to the within-group variation. A large F-value means that the between-group variation was greater than the within-group variation, which suggests a statistically significant difference in the group means.

#### 2.4.5. Classifier Model Construction and Evaluation

The support vector machines (SVMs) [31] with four different kernels (linear kernel, polynomial kernel, sigmoid kernel, and radial basis function (RBF) kernel), were evaluated and compared. Finally, the RBF kernel was selected as the kernel for all the classifiers deployed in this study. The kernel function is defined as:

(1) $$k\left(x_{i},x_{j}\right)=exp(-\gamma \cdot {\left\|x_{i}-x_{j}\right\|}^{2})$$

where $\gamma =1.0/(N_{feature}*var\left(x_{i}\right))$.

## 3. Results

### 3.1. Predicting LNM Using CT and FLIm

**Figure 2:**
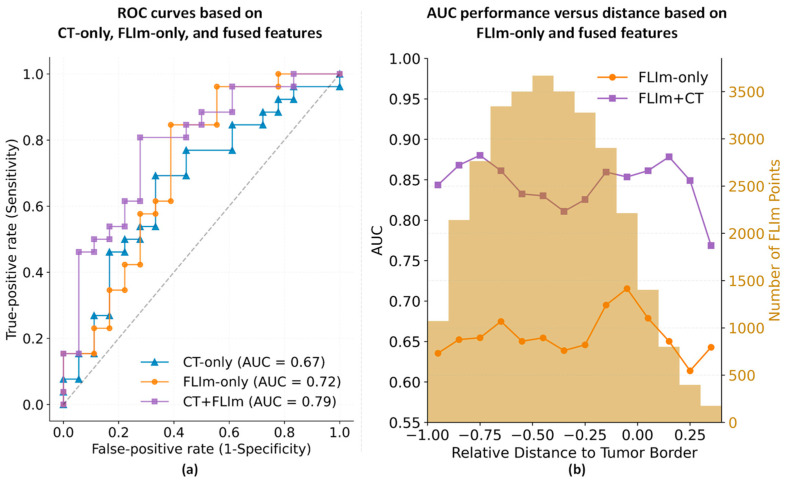
AUC comparisons using CT features only, FLIm features only and a combination of FLIm and CT features. (**a**) Comparison of ROC curves using fused features (purple curve), CT features alone (blue curve), and FLIm features alone (orange curve); (**b**) AUC performance versus relative distance from the location of the FLIm measurement to the tumor border based solely on fused features (purple curve) and FLIm features alone (orange curve), with yellow histograms illustrating the distribution in the relative distance of FLIm measurements. The relative distance is the ratio of the distance from the point to the tumor border (in centimeters) over the maximum distance within the tumor.

The resulting receiver operating characteristic (ROC) curves of the trained ML model using CT, FLIm, and combined features, respectively, are shown in Figure 2a. To evaluate the performance of the trained classifiers, we computed the overall testing balanced accuracies (bACC), as well as the areas under the ROC curves (AUC), using the surgical pathology as the ground truth. An AUC of 1.0 represents perfect prediction, while an AUC of 0.5 indicates no predictive ability, which is akin to random guessing. The bACC calculation is based on a default threshold of 0.5. The overall testing bACCs and the AUCs are listed in Table 2. The classifiers using CT + FLIm features achieved an average testing bACC of 0.65 ± 0.20 and AUC of 0.79, which outperformed the classifier using only FLIm features (testing bACC: 0.61 ± 0.30, AUC: 0.72) or only CT features (testing bACC: 0.58 ± 0.19, AUC: 0.67).

Given the potential for FLIm features to vary based on location within the tumor, Figure 2b plots the AUC performance of FLIm features as a function of the relative distance to the tumor border. These AUCs are overlaid on a yellow histogram depicting the distribution in the relative distance of all the measurements. The X-axis indicates the relative distance from the tumor border to the FLIm point, where distance = 0 indicates the position at the tumor border and distance = −1 indicates the most central position of the tumor. This relative distance is calculated as the ratio of the distance from the point to the tumor border over the maximum distance within the tumor, providing a normalized measure that allows for comparison across tumors of varying sizes. Negative values stand for the points located inside of the tumor. The left Y-axis denotes AUC performance while the right Y-axis indicates histogram counts.

As the spatial information was inferred under the presumption of tumors being elliptical, it resulted in some points being located outside the tumor’s confines, resulting in positive distances. However, since the number of points with a relative distance >0.4 was limited, they were removed in this specific analysis. As delineated in Figure 2b, when using combined features, central (relative distance between −1.0 and −0.7) and peripheral (relative distance between −0.2 and 0.2) points achieve slightly higher AUCs than intermediate points (relative distance between −0.6 and −0.25).

### 3.2. Feature Selection

**Figure 3:**
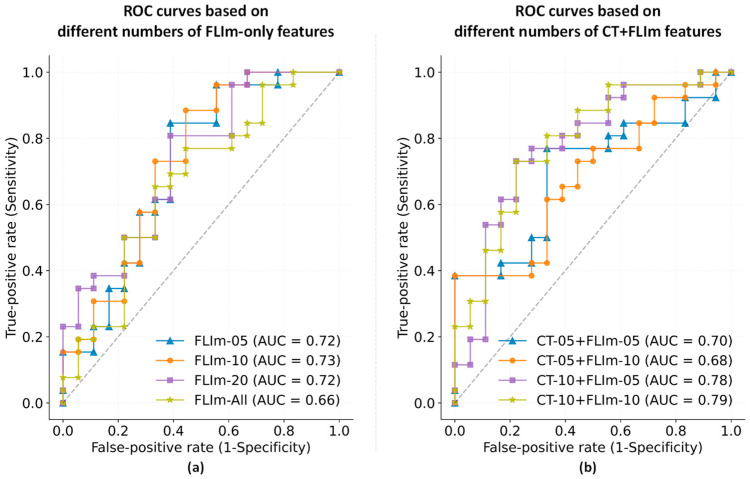
Comparative ROC curves for varying numbers of features. (**a**) FLIm-only classifiers: “FLIm-n” indicates that a total number of n FLIm features were selected. (**b**) CT + FLIm classifiers: “CT-n + FLIm-m” indicates that n CT features and m FLIm features were selected.

To balance CT and FLIm features and minimize overfitting issues, an initial feature selection was conducted. Figure 3a illustrates the ROC curves corresponding to varying numbers of FLIm features, e.g., 5, 10, and 20. Results indicated that a reduced FLIm feature set could outperform using the entire FLIm feature set. Specifically, a set of 5 features attained the highest AUC of 0.73, while sets of 10 and 20 features registered a marginally lower AUC of 0.71. To mitigate overfitting, we opted for 5 and 10 features, which were then advanced to the subsequent selection phase. To balance the CT and FLIm features, the CT feature count was also capped at 5 and 10. Figure 3b shows the results from different combinations of FLIm and CT features. The optimal AUC of 0.79 was achieved when using 10 FLIm features combined with 10 CT features (the yellow curve).

### 3.3. Performance in Relation to Tumor Size and Tumor Anatomical Site

**Figure 4:**
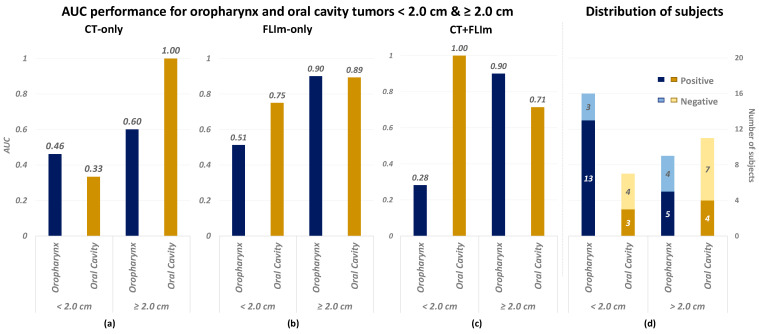
AUC performance and subject distribution for small (<2.0 cm) and large (≥2.0 cm) tumor dimensions spanning two tumor anatomical sites (oropharynx and oral cavity). (**a**) AUC for CT-only classification; (**b**) AUC for FLIm-only classification; (**c**) AUC for combined CT + FLIm classification; and (**d**) the distribution of subjects by LNM status across varying tumor dimensions and sites. The X-axis divides tumors based on anatomical location (oropharynx vs. oral cavity) and size (<2.0 cm vs. ≥2.0 cm).

To investigate the influence of tumor size and anatomical location on prediction accuracy, Figure 4a–c shows a comparison of AUC performance for three classifiers: (a) CT-only, (b) FLIm-only, and (c) CT + FLIm, each separated by tumor size and anatomical site (oropharynx versus oral cavity). The tumor size was determined by the greatest dimension extracted from the pathology report with a threshold set at 2.0 cm according to the AJCC cancer staging system [32]. Additionally, Figure 4d presents the distribution of positive and negative LNM cases among these tumor categories. Consistently, across Figure 4a–d, the X-axis segregates tumors by anatomical site and size. Notably, Figure 4a reveals that the CT-only classifier performs exceptionally well for tumors in the oral cavity measuring ≥2.0 cm; yet, it shows limited performance for smaller tumors and those in the oropharynx. In contrast, Figure 4b illustrates that the FLIm-only classifier maintains relatively high AUC values across most categories, with a notable exception for oropharynx tumors smaller than 2.0 cm. Figure 4c indicates varied AUC performance for the CT + FLIm classifier, with low values for small oropharynx tumors and large oral cavity tumors but higher values for large oropharynx tumors and small oral cavity tumors. This variability may suggest that tumor size could pose a diagnostic challenge.

## 4. Discussion

### 4.1. Overall

We have developed a combined FLIm–radiomic model capable of predicting LNM in HNC by utilizing primary tumor signatures derived from both CT and FLIm. By combining 42 FLIm features with 924 CT radiomic features, we employed an SVM model for LNM prediction. Our model demonstrated improved accuracy (testing bACC: 0.65, AUC: 0.79) compared to classifiers that used only FLIm or CT features. Further feature selection optimized the model, identifying a set of 10 FLIm and 10 CT features that provided optimal predictive capability. Additionally, our analysis highlighted the influence of tumor size and FLIm measurement location on prediction outcomes. This study underscores that combined pre-operative CT and intra-operative FLIm features of the primary tumor enhance both prediction accuracy and AUC, representing a marked improvement over using either CT or FLIm features alone.

Standard pretreatment radiographic staging, which includes pre-operative imaging with CT or other cross-sectional modalities, is routinely employed to evaluate the primary tumor site and assess the status of draining lymph nodes. Although pre-operative imaging provides valuable insights into the primary site and potential lymph node involvement, it often falls short in offering definitive assessments. Studies referenced in the introduction illuminate these challenges, pointing out the shortcomings of conventional imaging modalities in effectively differentiating between metastatic and benign lymphatic involvement. The limitations of current approaches highlight a critical gap in our ability to assess nodal disease status accurately. More specifically, while traditional methods, such as CT and MRI, have been widely used for lymph node evaluation, they often struggle to differentiate between inflammatory and metastatic nodes, particularly when node enlargement is minimal. Our study demonstrates that the combination of CT radiomics and FLIm offers improved prediction accuracy for LNM by leveraging both structural and functional information from the primary tumor. This approach, compared to standalone CT or MRI, may offer a more sensitive and non-invasive alternative, especially in cases where lymph nodes appear radiographically normal.

Our proposed method aims to overcome these limitations by leveraging optical imaging data, obtained intra-operatively during the surgical procedure with an emerging optical technology, intra-operative FLIm, which is undergoing clinical trials to evaluate its potential to enhance surgical assessments and improve negative margin rates during resection [33,34,35]. The methodology synergizes pre-operative and intra-operative data, merging CT and FLIm information of the primary tumor to complement each other. This can allow for the predictive assessment of malignant lymph nodes, with potential future implications for surgical planning and disease treatment. Our research indicates, as shown in Figure 2, that utilizing both CT and FLIm features improves the accuracy of malignant LNM detection over using either modality in isolation (AUC: 0.79 vs. 0.67/0.70). In addition, our feature contribution analysis revealed a preference for features derived from high-pass and low-pass wavelet-filtered 2D CT images and for the Laguerre coefficients from channel 3 (spectral band) of the FLIm signals. Detailed contribution scores are available in Supplementary Material Figure S4.

Compared to previously published works [36,37,38], the dataset size used in this study remains modest. To counteract the constraints of a limited dataset, a 10-fold nested CV was employed. This approach ensures reliable hyperparameter estimation and optimal usage of existing data to adjust hyperparameters and assess performance without overlap between these processes. Small datasets inherently exhibit fluctuating performance metrics based on specific train–test divisions. The nested CV, by aggregating performance over diverse splits, provides a more reliable evaluation of model performance. We are actively recruiting patients to further evaluate the robustness of our model.

### 4.2. Limitation

#### 4.2.1. Limited Performance for CT-Only Classifier

Based on the studies referenced above [36,37,38], the CT-only classifier exhibited lower bACC and AUC scores. The main rationale behind our approach was to concentrate solely on primary tumors, as our objective is to enhance the prediction accuracy of detecting radiographically normal LNMs. In contrast, other studies utilized features extracted from lymph nodes for classification purposes. Another contributing factor could be the heterogeneity of the CT scans used in our dataset. The CT data were sourced from over 15 institutions, utilizing scanners from various manufacturers, and encompassed a wide range of scanning protocols and reconstruction parameters. As a result, the CT images varied dramatically from patient to patient. This variability in CT protocols reflects the real-world diversity in clinical practice but also represents a limitation of the study, as it introduces potential heterogeneity in image quality and features. Although we applied the normalization recommended by the ISBI [25] to mitigate these differences, achieving consistency across the dataset proved to be a challenge. This variability is likely another reason why the CT-only classifier underperformed compared to those in other studies. However, it is important to note that the primary objective of our study was not to improve the performance of CT radiomic features per se, but rather to explore the benefits of integrating two different modalities, CT and FLIm data, over using one individual modality alone; therefore, the issues mentioned above do not affect the major conclusion of this study.

#### 4.2.2. Unbalanced Performance over Tumor Sizes and Anatomical Sites

Figure 4 illustrates the differential performance of various classifiers across tumor size and anatomical site, highlighting a distinct imbalance in diagnostic accuracy. In particular, the CT-only and combined CT + FLIm classifiers exhibited significant variability over different tumor categories. Different regions within HNC can not only show histological or morphological inconsistencies [39] but also reflect the varied tumor etiologies, such as those resulting from tobacco use versus human papilloma virus (HPV) infections [40,41]. Therefore, we conjecture that the information from different regions may also contribute differently to the prediction of malignant LNM. Additionally, we observed that the combined CT + FLIm classifier did not consistently outperform the individual single-modality classifiers across all tumor sizes and anatomical sites, for example, the AUC for CT + FLIm was lower than the separate analyses of CT and FLIm for tumors smaller than 2 cm. This inconsistency is potentially due to training on a composite dataset. However, similar to the issue mentioned in Section 4.2.1, the classifiers had to be trained on a composite dataset instead of stratified sub-datasets to avoid excessively small sample sizes, which would result in extremely poor performance for certain tumor categories.

Moving forward, the acquisition of a larger and more diverse dataset would present an opportunity to restructure our training approach. With more data, we aim to develop classifiers tailored to specific sub-categories of primary tumors defined by size and anatomical site. Training classifiers on these more focused sub-datasets could mitigate the issues observed with the mixed data approach. This targeted strategy is expected to strengthen the model’s capacity to discriminate between tumor characteristics with greater precision, thereby improving the overall diagnostic process for varying tumor presentations.

#### 4.2.3. Feature Fusion Method

In this study, we extracted radiomic features from pre-operative CT images and optical features from intra-operative FLIm, separately, then concatenated them for multimodal feature integration. We did not perform spatial registration between the FLIm measurement positions and the CT image due to (1) the lack of precise relative positions of surgical sites in juxtaposition with FLIm scan coordinates and (2) pronounced tissue deformation during the surgery, particularly around oral regions. Consequently, aligning images across modalities became arduous. Nonetheless, extracting radiomic features in the local region of the FLIm measurement may provide more site-specific information. In subsequent studies, our focus will shift towards surmounting these image registration challenges between FLIm and CT scans.

### 4.3. Future Perspectives

Looking ahead, the integration of multi-modal imaging techniques like CT and FLIm holds promise for improving the accuracy of LNM prediction and enabling more precise, individualized treatment planning. Future studies could explore the combination of these modalities with PET–CT or contrast-enhanced CT to further refine predictive models. In addition to multi-modal imaging, leveraging advanced machine learning techniques offers the potential for more robust predictive models. These models could better handle complex datasets, allowing for the integration of additional data types to form a more comprehensive and tailored diagnostic tool for clinical use.

## 5. Conclusions

We have developed a mixed FLIm-radiomic model adept at predicting LNM in HNC by harnessing both CT radiomics and FLIm-derived tumor signatures. Our study underscores that integrating pre-operative CT and intra-operative FLIm features enhances both prediction accuracy and AUC, demonstrating a marked improvement over relying on either CT or FLIm features in isolation.

## Figures and Tables

**Table 1:** Patient demographics, including sex, anatomy/primary tumor site, ratio of LNM, and age.

Demographic Data	Overall, in This Study
No. of patients	46
Sex	
Male	37 (80.4%)
Female	9 (19.6%)
Anatomy/primary tumor site	
Oral cavity	20 (43.5%)
Superior tongue	14(30.4%)
Gingiva	3 (6.5%)
Floor of mouth	2 (4.3%)
Palate	1 (2.2%)
Oropharyngeal cancer	26 (56.5%)
Palatine tonsil	14 (30.4%)
Base of tongue	7 (15.2%)
Pharynx	2 (4.3%)
Retrimolar trigone	2 (4.3%)
Vallecula	1 (2.2%)
Lymph node metastasis	
Positive	27 (58.7%)
Negative	19 (41.3%)
Age at surgery (y) *	63.3 ± 11.9
20~29	1 (2.2%)
30~39	1 (2.2%)
40~49	4 (8.7%)
50~59	10 (21.7%)
60~69	16 (34.8%)
70~79	11 (23.9%)
80~89	3 (6.5%)

Note: Except where other noted, data are numbers with percentages in parentheses. * Data are mean ± standard deviation.

**Table 2:** The testing bACCs and AUCs of different combinations of features.

	CT + FLIm	CT-Only	FLIm-Only
Test bACC	0.65	0.58	0.61
Test AUC	0.79	0.67	0.72

## Data Availability

The original contributions presented in the study are included in the article/Supplementary Material, further inquiries can be directed to the corresponding author/s.

## Supplementary Materials

The following supporting information can be downloaded at: https://www.mdpi.com/article/10.3390/diagnostics14182097/s1, Figure S1: The image examples. (a) CT images with different imaging protocols; (b) FLIm data; Figure S2. The schematic of the radiomic feature extraction and feature names. Shape-class features were extracted only from the original image; Figure S3: The schematic of dataset arrangement and nested cross-validation. The subjects were divided into ten subsets, D1 to D10, according to the rules of the conditional random grouping. For each subset of data, a classifier was trained using the nine remaining subsets of data; Figure S4: Heat maps of top 10 features for sub-groups. (a) CT features and (b) FLIm features. The first two columns are the feature index and the name. The columns “CF0–CF9” indicate the classifiers of 10 groups. The “counts” column is the total number of times that the feature was selected in the top 10 features. The last column, “averaging”, is the averaged score over all groups. (0 value in the heat maps indicate that the feature was not ranked in the top 10 features, not that the score was 0).
