# Supplementary material for: Early Detection of Lymph Node Metastasis Using Primary Head and Neck Cancer Computed Tomography and Fluorescence Lifetime Imaging
Source: Diagnostics (Basel). 2024 Sep 23;14(18):2097. doi: 10.3390/diagnostics14182097 (PMC11430879; doi:10.3390/diagnostics14182097)
Supplement: Supplementary file 1 [file diagnostics-14-02097-s001.zip › diagnostics-3205938-supplementary.pdf]

diagnostics MDPI

Supplementary Materials

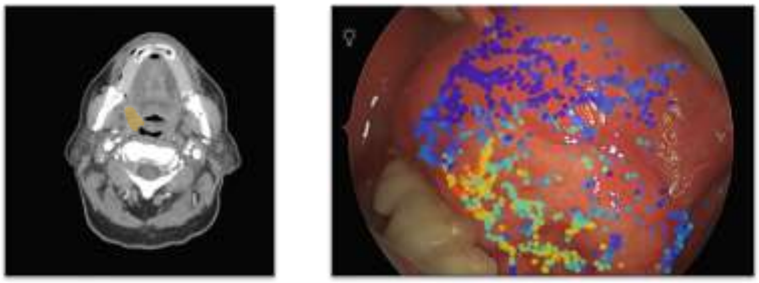

(a) CT examples **(b) FLIm examples**

Figure S1. The image examples. (a) CT images with different imaging protocols; (b) FLIm data.

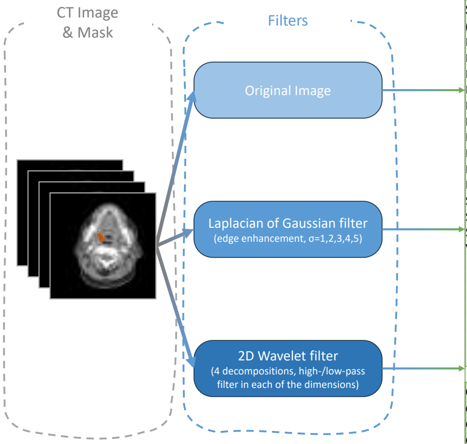

Features

**Shape (14 features)**	**First Order (18 features)**	**GLCM (22 features)**	**GLRLM (16 features)**
**(computed for original image only)**	10Percentile	Autocorrelation	GrayLevelNonUniformity
Elongation	90Percentile	JointAverage	GrayLevelNonUniformityNormalized
Flatness	Energy	ClusterProminence	GrayLevelVariance
LeastAxisLength	Entropy	ClusterShade	HighGrayLevelRunEmphasis
MajorAxisLength	InterquartileRange	ClusterTendency	LongRunEmphasis
Maximum2DDiameterColumn	Kurtosis	Contrast	LongRunHighGrayLevelEmphasis
Maximum2DDiameterRow	Maximum	Correlation	LongRunLowGrayLevelEmphasis
Maximum2DDiameterSlice	MeanAbsoluteDeviation	DifferenceAverage	LowGrayLevelRunEmphasis
Maximum3DDiameter	Mean	DifferenceEntropy	RunEntropy
MeshVolume	Median	DifferenceVariance	RunLengthNonUniformity
MinorAxisLength	Minimum	JointEnergy	RunLengthNonUniformityNormalized
Sphericity	Range	JointEntropy	RunPercentage
SurfaceArea	RobustMeanAbsoluteDeviation	Imc1	RunVariance
SurfaceVolumeRatio	RootMeanSquared	Imc2	ShortRunEmphasis
VoxelVolume	Skewness	Idm	ShortRunHighGrayLevelEmphasis
	TotalEnergy	Idmn	ShortRunLowGrayLevelEmphasis
	Uniformity	Id	
	Variance	Idn	
		InverseVariance	
		MaximumProbability	
		SumEntropy	
		SumSquares	
**GLSZM (16 features)**	**GLDM (14 features)**	**NGTDM (5 features)**	
GrayLevelNonUniformity	DependenceEntropy	Busyness	
GrayLevelNonUniformityNormalized	DependenceNonUniformity	Coarseness	
GrayLevelVariance	DependenceNonUniformityNormalized	Complexity	
HighGrayLevelZoneEmphasis	DependenceVariance	Contrast	
LargeAreaEmphasis	GrayLevelNonUniformity	Strength	
LargeAreaHighGrayLevelEmphasis	GrayLevelVariance		
LargeAreaLowGrayLevelEmphasis	HighGrayLevelEmphasis		
LowGrayLevelZoneEmphasis	LargeDependenceEmphasis		
SizeZoneNonUniformity	LargeDependenceHighGrayLevelEmphasis		
SizeZoneNonUniformityNormalized	LargeDependenceLowGrayLevelEmphasis		
SmallAreaEmphasis	LowGrayLevelEmphasis		
SmallAreaHighGrayLevelEmphasis	SmallDependenceEmphasis		
SmallAreaLowGrayLevelEmphasis	SmallDependenceHighGrayLevelEmphasis		
ZoneEntropy	SmallDependenceLowGrayLevelEmphasis		
ZonePercentage			
ZoneVariance			

Figure S2. The schematic of the radiomic feature extraction and feature names. Shape-class features were extracted only from the original image.

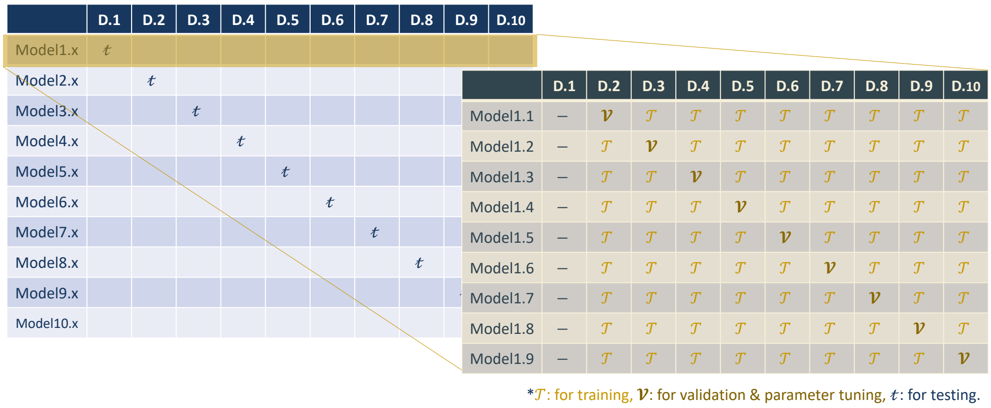

Figure S3. The schematic of dataset arrangement and nested cross-validation. The subjects were divided into 10 subsets, D1 to D10, according to the rules of the conditional random grouping. For each subset of data, a classifier was trained using the 9 remaining subsets of data.

Ind.	Feature Name	CF0	CF1	CF2	CF3	CF4	CF5	CF6	CF7	CF8	CF9	Counts	Averaging
51	original_shape_Maximum2DDiameterSlice	0	0	7.15	0	0	0	5.51	0	0	0	2	1.266
67	original_firstorder_Mean	0	0	0	0	0	7.71	0	0	0	0	1	0.771
68	original_firstorder_Median	0	0	0	0	0	7.52	0	0	0	0	1	0.752
73	original_firstorder_Skewness	0	6.17	0	7.97	6.82	7.55	0	0	0	0	4	2.851
236	log-sigma-1-0-mm-3D_ngtdm_Busyness	0	0	7.2	0	0	0	0	0	0	0	1	0.72
303	log-sigma-2-0-mm-3D_glszm_LargeAreaLowGrayLevelEmphasis	0	0	6.46	6.21	0	0	0	0	0	0	2	1.267
**544**	**log-sigma-5-0-mm-3D_glcm_Imc1**	7.42	6.5	6.56	9.66	8.04	9.02	0	0	0	6.64	7	5.384
545	log-sigma-5-0-mm-3D_glcm_Imc2	0	0	0	0	0	0	5.58	0	0	0	1	0.558
697	wavelet-HL_firstorder_90Percentile	0	0	0	0	6.82	0	0	7.08	0	0	2	1.39
**704**	**wavelet-HL_firstorder_Mean**	6.77	7.17	8.58	10.31	11.93	13.49	6.6	10.5	8.84	9.44	10	9.363
705	wavelet-HL_firstorder_Median	0	0	0	6.44	10.58	8.89	0	0	0	0	3	2.591
**709**	**wavelet-HL_firstorder_RootMeanSquared**	7.68	7.97	9.37	11.35	12.66	14.05	7.5	11.83	10.24	10.57	10	10.322
**710**	**wavelet-HL_firstorder_Skewness**	0	0	7.36	9.67	6.66	8.46	6.42	7.26	0	0	6	4.583
**713**	**wavelet-HL_firstorder_Variance**	6.24	5.15	0	0	0	0	0	7.29	9.35	7.09	5	3.512
**716**	**wavelet-HL_glcm_ClusterProminence**	7.13	5.31	5.87	0	0	0	5.36	0	8.84	7.89	6	4.04
**717**	**wavelet-HL_glcm_ClusterShade**	7.57	9.84	13.49	12.31	11.65	11.55	10.01	13.41	12.95	11.54	10	11.432
718	wavelet-HL_glcm_ClusterTendency	0	0	0	0	0	0	0	0	8.28	0	1	0.828
719	wavelet-HL_glcm_Contrast	5.99	0	0	0	0	0	0	0	7.58	0	2	1.357
723	wavelet-HL_glcm_DifferenceVariance	6.72	0	0	0	0	0	0	0	0	0	1	0.672
732	wavelet-HL_glcm_InverseVariance	0	0	0	0	0	0	0	7.04	0	0	1	0.704
735	wavelet-HL_glcm_SumSquares	0	0	0	0	0	0	0	0	8.3	0	1	0.83
738	wavelet-HL_glrlm_GrayLevelVariance	0	0	0	0	0	0	0	0	8.4	6.75	2	1.515
761	wavelet-HL_glszm_SizeZoneNonUniformityNormalized	0	0	0	0	6.08	0	0	0	0	0	1	0.608
**773**	**wavelet-HL_gldm_GrayLevelVariance**	6.26	5.19	0	0	0	0	5.34	7.32	9.37	7.1	5	4.058
**784**	**wavelet-HL_ngtdm_Complexity**	6.75	0	6.89	5.72	0	0	5.52	7.16	0	8.27	6	4.031
886	wavelet-LL_firstorder_Mean	0	0	0	0	0	7.55	0	0	0	0	1	0.755
892	wavelet-LL_firstorder_Skewness	0	5.72	0	7.25	7.38	0	0	0	0	0	3	2.035
915	wavelet-LL_glcm_MaximumProbability	0	4.74	0	0	0	0	6.17	8.87	0	8.31	4	2.809

(**a**)

Ind.	Feature Name	CF0	CF1	CF2	CF3	CF4	CF5	CF6	CF7	CF8	CF9	Counts	Averaging
**0**	**lifet_avg_ch1**	1111.63	1523.60	1112.50	0	1085.29	557.93	1182.74	0	1573.13	992.35	8	913.918
1	lifet_avg_ch2	0	0	0	0	0	533.04	0	0	0	0	1	53.304
**2**	**lifet_avg_ch3**	1062.96	1504.31	1250.83	1270.82	2147.96	0	2642.23	3109.29	0	1137.81	8	1412.620
3	spec_int_ch1	0	0	0	0	0	751.76	0	0	0	0	1	75.176
4	spec_int_ch2	0	0	0	0	0	738.35	0	0	0	0	1	73.835
5	spec_int_ch3	0	0	0	0	0	665.77	0	0	0	0	1	66.577
6	Laguerre_coeffs_1_ch1	0	0	0	0	0	598.69	0	0	0	0	1	59.869
**7**	**Laguerre_coeffs_2_ch1**	2650.21	3068.25	3108.07	2193.13	3105.42	1639.18	2326.97	3614.40	4061.92	2403.00	10	2817.054
**9**	**Laguerre_coeffs_4_ch1**	1697.81	2148.45	1983.42	1472.10	1913.63	881.28	1371.57	2124.17	2445.62	1494.31	10	1753.235
11	Laguerre_coeffs_6_ch1	0	1150.42	0	0	0	0	0	0	1199.19	0	2	234.961
**31**	**Laguerre_coeffs_2_ch3**	2336.91	2681.08	2569.04	2016.74	2768.36	1005.26	3317.20	3993.64	1589.04	1970.89	10	2424.815
**33**	**Laguerre_coeffs_4_ch3**	2315.11	2797.82	2611.79	2241.95	2880.21	791.10	3150.71	4134.16	1842.86	2102.32	10	2486.802
**35**	**Laguerre_coeffs_6_ch3**	1906.63	2406.35	2214.43	2098.38	2540.26	0	2632.92	3655.85	1762.81	1848.34	9	2106.599
**37**	**Laguerre_coeffs_8_ch3**	1658.48	2091.64	1902.73	1946.76	2243.39	0	2280.73	3214.03	1624.04	1636.43	9	1859.822
**39**	**Laguerre_coeffs_10_ch3**	1358.58	1730.33	1556.78	1664.88	1832.68	0	1821.66	2755.90	1390.41	1435.11	9	1554.634
40	Laguerre_coeffs_11_ch3	0	0	0	948.91	0	0	0	1301.74	0	0	2	225.064
**41**	**Laguerre_coeffs_12_ch3**	836.17	0	980.95	1080.82	1117.55	0	1096.06	1879.16	895.40	1023.41	9	890.952

(**b**)

Figure S4. Heat maps of top-10 features for sub-groups. (a) CT features and (b) FLIm features. The first two columns are the feature index and the name. The columns "CF0~CF9" indicate the classifiers of 10 groups. The "counts" column is the total number of times that the feature was selected in the top-10 features. The last column "Averaging" is the averaged score over all groups. (0 value in the heat maps indicate that the feature was not ranked in the top-10 features not that the score was 0.)
